# Balancing Selection Drives the Maintenance of Genetic Variation in *Drosophila* Antimicrobial Peptides

**DOI:** 10.1093/gbe/evz191

**Published:** 2019-09-04

**Authors:** Joanne R Chapman, Tom Hill, Robert L Unckless

**Affiliations:** Department of Molecular Biosciences, University of Kansas

**Keywords:** antimicrobial peptides (AMPs), host-defense peptides (HDPs), population genetics, immunity, coevolution, *Sophophora*

## Abstract

Genes involved in immune defense against pathogens provide some of the most well-known examples of both directional and balancing selection. Antimicrobial peptides (AMPs) are innate immune effector genes, playing a key role in pathogen clearance in many species, including *Drosophila*. Conflicting lines of evidence have suggested that AMPs may be under directional, balancing, or purifying selection. Here, we use both a linear model and control-gene-based approach to show that balancing selection is an important force shaping AMP diversity in *Drosophila*. In *Drosophila melanogaster*, this is most clearly observed in ancestral African populations. Furthermore, the signature of balancing selection is even more striking once background selection has been accounted for. Balancing selection also acts on AMPs in *Drosophila mauritiana*, an isolated island endemic separated from *D. melanogaster* by about 4 Myr of evolution. This suggests that balancing selection may be broadly acting to maintain adaptive diversity in *Drosophila* AMPs, as has been found in other taxa.

## Introduction

Pathogens exert strong selective pressures on their hosts, both in terms of individual fitness and the evolutionary trajectory of populations and species (Anderson and May 1981). Coevolutionary dynamics of hosts and pathogens results in continual selection for adaptive improvements in both players, often referred to as a coevolutionary arms race (Paterson et al. 2010; Schulte et al. 2010; Thrall et al. 2012). As a consequence, genes involved in immune defense tend to undergo strong positive selection, such that they are among the fastest evolving genes in the genomes of many hosts (Nielsen et al. 2005; Kosiol et al. 2008; Downing et al. 2009a; Ekblom et al. 2010; McTaggart et al. 2012).

However, resistance mutations may not always become fixed. Balancing selection is the process whereby polymorphism is adaptively maintained over extended timescales, sometimes described as trench-warfare dynamics (Stahl et al. 1999). Several processes are thought to contribute to balancing selection (reviewed by Llaurens et al. [2017]). These include heterozygote advantage, whereby individuals heterozygous at a given locus have a fitness advantage over either homozygote; negative frequency-dependent selection, whereby the benefit of an allele increases the rarer it is in a population; and selection varying in a context-dependent manner, for example, at different spatial or temporal scales, between the sexes, or in the presence or absence of infection. Balancing selection can be detected as an excess of intermediate frequency variants and a region of increased polymorphism around the selected site.

The extent to which selection will impact genetic variation within and around immune genes will depend on a number of factors. These include the form and strength of selection (Kingsolver and Pfennig 2007); the genetic architecture (e.g., dominance, epistasis) of immune traits (Mackay 2001); the timescale upon which selection is acting (Charlesworth 2006); the density, diversity, and virulence of pathogens (Lambrechts et al. 2006); the cost of maintaining resistance alleles in the absence of infection (Unckless and Lazzaro 2016); effective population size (Charlesworth 2009); the mutation and recombination rates of hosts and pathogens (Gandon and Michalakis 2002); environmental variables (Wolinska and King 2009); and demographic factors such as gene flow and bottlenecks (Brockhurst et al. 2003).

The dynamic selective pressures exerted by pathogens promote balanced polymorphism of host immune genes in several cases. Perhaps the best documented example is the major histocompatibility complex (MHC) in vertebrates (reviewed in Hughes and Yeager [1997], Edwards and Hedrick [1998], Hedrick [1998], and Bernatchez and Landry [2003]). Individuals tend to be heterozygous at MHC loci, and large numbers of MHC alleles are maintained in populations. Other examples of balancing selection acting on host immune genes in animals include Toll-like receptors in humans (Ferrer-Admetlla et al. 2008), deer (Quéméré et al. 2015, 2018), bank voles (Kloch et al. 2018), and birds (Alcaide and Edwards 2011; Gilroy et al. 2017; Velová et al. 2018); various cytokine genes (particularly interleukins) in humans (Hughes et al. 2005; Wilson et al. 2006; Ferrer-Admetlla et al. 2008; Fumagalli et al. 2009), birds (Downing et al. 2009a, 2009b, 2010), and field voles (Turner et al. 2012); and viral resistance genes including *Oas1b* in mice (Ferguson et al. 2008), *OAS1* in primates (Ferguson et al. 2012; Fish and Boissinot 2015), and *TRIM5* in humans (Cagliani et al. 2010) and primates (Newman et al. 2006).

Balancing selection also appears to play a role in the evolution of antimicrobial peptides (AMPs) in some taxa. AMPs are effectors of innate immunity that are strongly induced upon infection (Lemaitre et al. 1997; Tzou et al. 2000). They are often membrane active (Shai 2002; Brogden 2005), with a direct role in killing and/or impeding the growth of pathogens (De Gregorio et al. 2002; Lemaitre and Hoffmann 2007). Balancing selection has been implicated as a driver of AMP evolution in a diverse array of species including birds (Hellgren and Sheldon 2011; Chapman et al. 2016), amphibians (Tennessen and Blouin 2008), fish (Halldórsdóttir and Árnason 2015), mollusks (Gosset et al. 2014), and humans (Cagliani et al. 2008; Hollox and Armour 2008).

The fruit fly, *Drosophila melanogaster*, is an important model for understanding evolution of the immune system (Hultmark 1993; Hoffmann 2003; Janssens and Beyaert 2003; Dostert et al. 2005; Lamiable et al. 2016). Directional selection on *Drosophila* immune genes appears to be a relatively widespread phenomenon, especially among antiviral, receptor, and signaling genes (Schlenke and Begun 2003; Obbard et al. 2006, 2009; Clark et al. 2007; Heger and Ponting 2007; Sackton et al. 2007; Hill et al. 2019). In contrast, evidence for balancing selection acting on *Drosophila* immune genes has been more equivocal. Genome-wide scans by Croze et al. (2016, 2017) found little evidence for balancing selection acting on immune genes. In contrast, both single gene and genome-wide analyses of selection have indicated that balancing selection may play an important role in the evolution of AMPs in *Drosophila* (Hanson et al. 2016; Unckless and Lazzaro 2016; Unckless et al. 2016). One striking example is the AMP Diptericin. This AMP is subject to balancing selection in *D. melanogaster*, likely driven by a tradeoff between immune defense and another life-history trait (Unckless et al 2016). The same balanced polymorphism, achieved via a different mutation to the derived allele, is found in *Drosophilasimulans* (Unckless et al 2016). Additionally, recent analyses have shown that both spatial and temporal fluctuations are associated with variation in *D. melanogaster* allele frequencies (Bergland et al. 2014), particularly in immune genes, including AMPs (Bergland et al. 2014; Early et al. 2017; Behrman et al. 2018).

AMPs play a key role in controlling pathogen load and infection outcome (De Gregorio et al. 2002; Lemaitre and Hoffmann 2007), which may be particularly important for insects and other invertebrates that lack an adaptive immune system. Given their direct interaction with pathogens, it is surprising that insect AMPs often do not show signatures of recurrent adaptive substitutions. We hypothesize that AMPs in *Drosophila* are prone to balancing selection. To test this hypothesis, we examined patterns of nucleotide variation at AMP, immune-, and control-gene loci in four populations of *D.melanogaster* and one population of *Drosophila mauritiana*. Using both a linear model and a matched control-gene-based approach, with standard population genetic statistics, we searched for molecular evolutionary signatures of selection on AMPs and immune genes. Our results provide evidence that balancing selection is an important driver of AMP evolution.

## Results

### Genetic Variation across Four *D. melanogaster* Populations

To determine whether AMPs show signatures of balancing selection, we obtained coding sequence alignments for 13,494 genes (including 35 AMPs and 288 genes putatively involved in immune defense; hereafter: immune genes) (Lack et al. 2016) for four *D. melanogaster* populations: Zambia (ZI), Rwanda (RG), France (FR), and North Carolina (DGRP) and quantified nucleotide polymorphism (supplementary table S1, Supplementary Material online). *Drosophilamelanogaster* originated in sub-Saharan Africa, expanded into Europe ∼15–16,000 years ago, and subsequently spread to North America <200 years ago (David and Capy 1988; Li and Stephan 2006; Keller 2007). The ZI and RG lines therefore represent ancestral populations, whereas FR and DGRP are derived populations. For each autosomal gene, we calculated three population genetic statistics: Watterson’s *θ* (the number of segregating sites, corrected for sample size), *π* (pairwise nucleotide diversity), and Tajima’s *D* across all populations, for silent (four-fold degenerate) sites, per after controlling for missing data. We limited our analyses to silent sites to allow us to reduce the possibility that our results were due to relaxed constraint on nonsynonymous variation rather than balancing selection. Relaxed constraint at the protein level should increase nonsynonymous diversity because selection against amino acid changes is lowered (Hartl and Clark 2006; Wang et al. 2016). Excluding nonsynonymous sites therefore allows us to specifically focus on the footprint of balancing selection. We then grouped genes as AMPs, immune genes, and background genes. The mean Tajima’s *D* for AMPs is higher than the mean of background genes in all populations (ZI, −0.284 AMPs vs. −0.874 autosomal average; RG, −0.110 vs. −0.232; FR, −0.064 vs. −0.113; DGRP, −0.041 vs. −0.596, supplementary table S2, Supplementary Material online), consistent with relatively more balancing selection occurring in AMPs. As observed previously (e.g., Glinka et al. 2003; Shapiro et al. 2007), the autosome-wide average for Tajima’s *D* is quite negative in *D. melanogaster*, which likely reflects a complex demographic history (supplementary fig. S1, Supplementary Material online).

As selection across the genome can be affected by differing levels of mutation and recombination, we next tested for differences in population genetic statistics between AMPs and the autosomal background after controlling for genomic position. We specifically tested whether AMPs have higher values of the three population genetic statistics by employing a linear model with four covariates: gene length, chromosome, chromosomal region (nested in chromosome, explained in more detail in the Materials and Methods), and gene type (AMP or not, nested in chromosomal region and chromosome). This revealed that population genetic measures were elevated for AMPs in ancestral populations (ZI and RG), but not derived populations (DGRP and FR) (table 1 and supplementary table S3 and supplementary fig. S1, Supplementary Material online).

**Table 1:** Linear Model for Various Population Genetic Statistics (Tajima’s *D*, *π*, and Watterson’s *θ* [*θ*_w_]) Suggests AMPs Are Elevated, Consistent with Balancing Selection in Several *Drosophila* Populations

Pop.	Stat.	AMP (*F*/*P*)	Region (*F*/*P*)	Length (*F*/*P*)	Chr (*F*/*P*)
	df	12	9	1	2
DGRP	*π*	1.24/0.254	5.32/**<0.001**	1.18/0.278	6.35/**0.002**
	*θ*_w_	3.14/**<0.001**	5.56/**<0.001**	0.682/0.410	3.38/**0.035**
	*D*	0.88/0.565	1.25/0.267	0.17/0.681	5.37/**0.005**
FR	*π*	0.73/0.714	5.50/**<0.001**	21.17/**<0.001**	1.05/0.350
	*θ*_w_	3.87/**<0.001**	4.38/**<0.001**	28.23/**<0.001**	1.85/0.16
	*D*	1.68/0.077	5.31/**<0.001**	0.05/0.833	1.03/0.357
RG	*π*	3.56/**<0.001**	5.90/**<0.001**	1.07/0.302	3.14/**0.045**
	*θ*_w_	3.33/**<0.001**	6.55/**<0.001**	1.02/0.313	3.43/**0.034**
	*D*	1.39/0.169	2.99/**0.002**	0.11/0.744	0.14/0.871
ZI	*π*	2.77/**0.001**	5.52/**<0.001**	0.93/0.336	3.53/**0.030**
	*θ*_w_	1.84/**0.042**	6.31/**<0.001**	0.77/0.380	4.55/**0.011**
	*D*	2.70/**0.002**	3.15/**0.001**	0.49/0.484	0.18/0.837
	df	11	8	1	2
*D. mau*	*π*	1.55/0.117	3.48/**<0.001**	0.17/0.682	5.49/**0.005**
	*θ*_w_	1.71/0.072	4.09/**<0.001**	0.26/0.608	14.00/**<0.001**
	*D*	1.56/0.113	2.12/**0.035**	0.54/0.463	5.23/**0.006**

Note.—These linear models include only genes within 100,000 bp and within ten times the size of an antimicrobial peptide. Data is presented as *F*-statistic/*P*-value from the linear model, with degrees of freedom (df) denoted in the second header row. *P* values <0.05 are in bold. AMP refers to AMP nested in region nested in chromosome and region refers to region nested in chromosome. Linear models were run individually for five *Drosophila* populations: four *D. melanogaster* populations (DGRP, *Drosophila* Genetics Reference Panel from North Carolina, USA; FR, France; RG, Rwanda; ZI, Zambia), and one *D. mauritiana* (*D. mau*) population. All three statistics (Tajima's *D*, π and θ_W_) were calculated on silent (four-fold degenerate) sites only.

### AMP-Control Tests for Balancing Selection in *Drosophila*

**Fig. 1.**
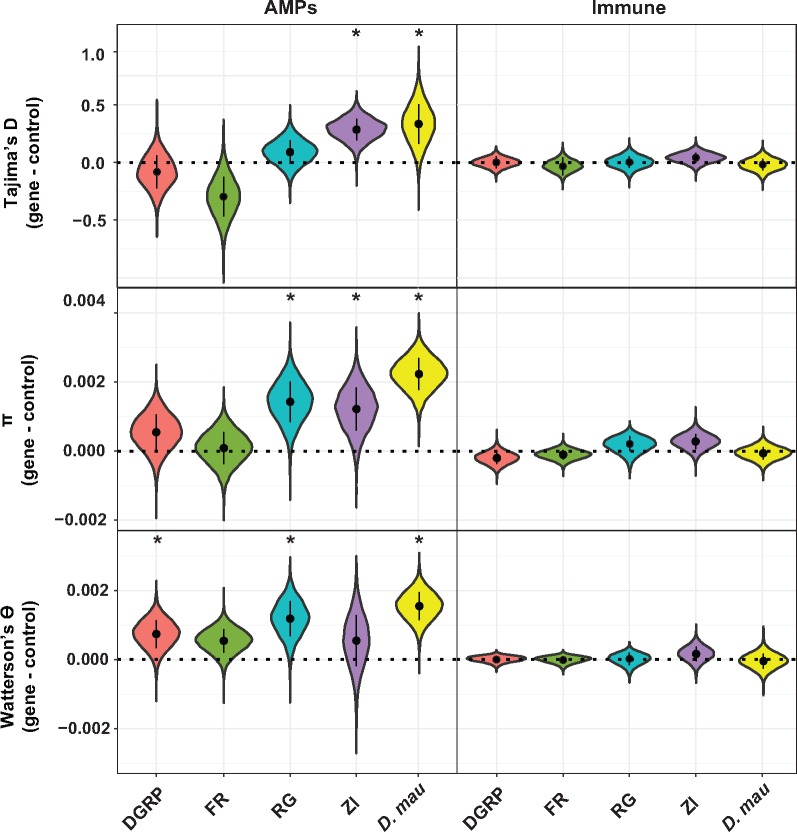
—Overall, AMPs show more evidence for balancing selection than other immune genes. Difference in means between 35 AMPs and randomly chosen control genes (left-hand side) or 288 immune genes and randomly chosen control genes (right-hand side), resampled 10,000 times, separated by population (DGRP = *Drosophila* Genetics Reference Panel from North Carolina, USA; FR = France; RG = Rwanda; ZI = Zambia; *D. mau*, *D. mauritiana*). Top panel: Tajima’s *D*; middle panel: *π* (nucleotide diversity); bottom panel: Watterson’s *θ*. All three statistics were calculated on silent (four-fold degenerate) sites only. The black dot within each plot shows the median for that population, and the black bar around the dot visualizes the interquartile range of the distribution. Values above 0 are consistent of balancing selection. Asterisks indicate cases where <5% of resamplings have values <0.

Given the apparent differences in selection between AMPs and the background averages described above, we also employed an AMP-control approach to test whether AMPs showed elevated diversity (a signature of balancing selection) in *D. melanogaster* and *D. mauritiana* while controlling for local variation in mutation and recombination rates. This approach also allowed us to visualize the differences found in the linear models above. For each AMP, we randomly sampled genes of similar length (coding sequence length ≤10 times the size of the AMP) and genomic position (within 100,000 bp on either side), calculated statistics for the AMP and control gene, and then calculated the mean difference over the 35 AMP/control comparisons. We repeated this procedure 10,000 times to obtain an empirical distribution of differences (fig. 1). Each AMP was associated with 8–30 control genes, resulting in each replicate containing a unique set of control genes. In these instances, a positive difference suggests a higher value for AMPs versus the control gene, consistent with balancing selection. Indeed, the differences are primarily positive for both *π* and Watterson’s *θ* for all populations (fig. 1*B* and *C* and table 2). For Tajima’s *D*, the differences are positive for Zambia and Rwanda (ancestral populations), consistent with balancing selection, but close to 0 for France and negative for DGRP (derived populations, fig. 1*A* and table 2). In line with our previous analyses, we found that AMPs had higher Tajima’s *D* in both RG and ZI, but not FR or DGRP. These results were recapitulated when we subsampled AMPs to control for the fact that they often cluster in the genome (see Materials and Methods, supplementary fig. S2, Supplementary Material online). We also examined all non-AMP immune genes using this control gene method and found little evidence of balancing selection in immune genes as a whole, in general concordance with Croze et al. (2017, 2016) (fig. 1 and supplementary table S4, Supplementary Material online).

**Table 2:** Control Gene Comparisons Suggest AMPs Are Subject to Balancing Selection, Particularly in Ancestral Populations

AMP − Control Statistics	DGRP	FR	RG	ZI	*D. mau*
Tajima’s *D* diff. > 0 (%)	28.7	4.1	81.4	99.7	98.1
Tajima’s *D* Mean diff.	−0.084	−0.295	0.092	0.289	0.26
Tajima’s *D* diff. std. dev.	0.142	0.171	0.102	0.092	0.12
*π* diff. > 0 (%)	85.9	58.4	98.9	96.9	100
*π* mean diff.	9.5 × $10^{-5}$	9.6 × $10^{-5}$	1.4 × $10^{-3}$	1.2 × $10^{-3}$	1.2 × $10^{-5}$
*π* diff. std. dev.	5.5 × $10^{-4}$	4.8 × $10^{-5}$	5.5 × $10^{-3}$	6.1 × $10^{-4}$	2.1 × $10^{-6}$
*θ*_w_ diff. > 0 (%)	96.2	93.7	98.5	77.4	99.9
*θ*_w_ mean diff.	7.5 × $10^{-4}$	5.5 × $10^{-4}$	1.2 × $10^{-3}$	5.6 × $10^{-4}$	1.7 × $10^{-5}$
*θ*_w_ diff. std. dev.	4.1 × $10^{-4}$	3.4 × $10^{-4}$	5.1 × $10^{-4}$	7.4 × $10^{-4}$	1.5 × $10^{-6}$

Note.—AMP minus control gene differences for three statistical measures (Tajima’s *D*, *π*, and Watterson’s *θ* [*θ*_w_]) of selection in four *D. melanogaster* populations (DGRP, *Drosophila* Genetics Reference Panel from North Carolina, USA; FR, France; RG, Rwanda; ZI, Zambia), and one *D. mauritiana* (*D. mau*) population. First row per statistic: percentage (%) of 10,000 replicates in which the AMP minus control difference (diff.) was positive (>0), suggestive of balancing selection; second row: mean AMP minus control difference across 10,000 replicates; third row: standard deviation (std. dev.) of the mean. All three statistics (Tajima's *D*, π and θ_W_) were calculated on silent (four-fold degenerate) sites only.

### Accounting for Background Selection Strengthens the Signature of Balancing Selection on *Drosophila* AMPs

**Fig. 2.**
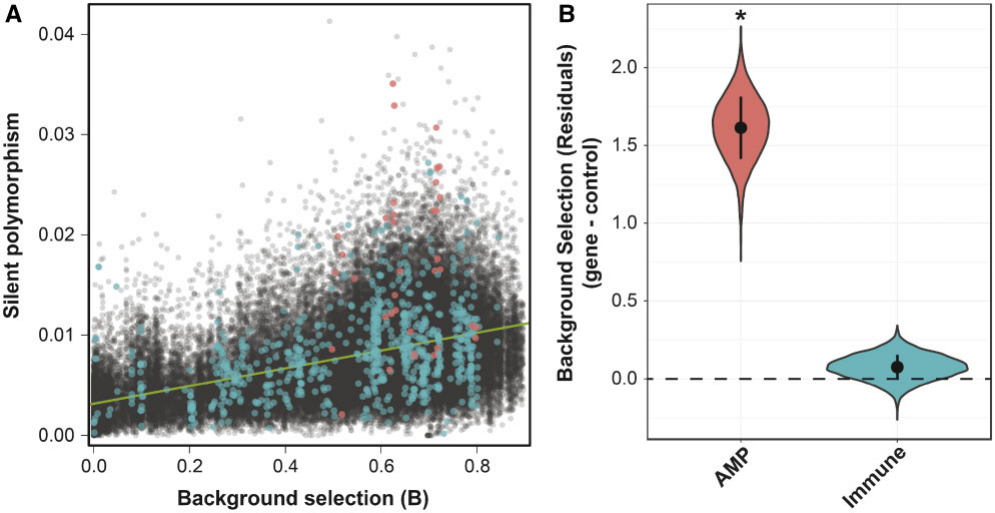
—Accounting for background selection in the Rwanda (RG) population strengthens the signal of balancing selection on AMPs. (*A*) Correlation between silent polymorphism and the background selection statistic (*B*) in 1,000-bp windows for the Rwanda population of *D. melanogaster*. The line of best fit is in blue and regions containing AMPs are indicated by red dots. (*B*) Resampling of mean difference (AMP/immune minus control) in the residuals after regressing silent polymorphism against the background selection statistic *B*. Values above 0 are consistent of balancing selection. Asterisks indicate cases where <5% of resamplings have values <0.

Background selection, the removal of neutral variation due to selection against linked deleterious alleles, can influence levels of polymorphism across the genome. Comeron (2014) calculated the observed amount of background selection across the genome in 1,000-bp windows in the *D. melanogaster* Rwanda population. He then correlated silent polymorphism against this measure. Regions with positive residuals (more silent polymorphism than expected based on background selection) were deemed to be under balancing selection, whereas those with negative residuals (less silent polymorphism than expected based on background selection) were deemed to be under directional selection. Two regions that contain AMPs (IM4 and Cecropin) were among the handful of outliers discussed by Comeron (2014) as being under balancing selection, which further motivated us to examine the general pattern for AMPs. We identified all AMP-containing windows and replotted Comeron’s data. This revealed that AMPs tend to fall in regions well above the trend-line (pink points, fig. 2*A*), indicating they are, in general, evolving in a manner consistent with balancing selection. In contrast, immune genes do not show elevated residuals compared with neighboring genes (teal points, fig. 2*A*). To further ascertain whether, as a group, AMPs show signatures of balancing selection, we used Comeron’s background selection data (Comeron 2014) to fit a linear model as described above but also included Comeron’s M1 statistic for background selection for a particular region as a covariate. In this case, AMPs showed significantly elevated silent polymorphism compared with other genes, whether we looked genome wide (*F*_10, 41533_=16.66, *P* < 0.0001) or focused on 100,000-bp regions of the genome containing AMPs (F_10, 1288_=10.59, *P* < 0.0001). We also found that regions containing AMPs, but not immune genes, were significantly elevated for residuals compared with nearby regions using our resampling approach (fig. 2*B*). This supports Comeron’s assertion that accounting for background selection improves the ability to detect balancing selection (Comeron 2014) and also supports our previous results showing that AMPs as a group are likely subject to balancing selection.

### Balancing Selection Also Acts on *D. mauritiana* AMPs

We also calculated population genetic statistics for 9,980 genes in 107 *D. mauritiana* isofemale lines, sequenced as a pool (Nolte et al. 2013). *D. mauritiana* is an island endemic which diverged from *D. melanogaster* ∼3–5 Ma (Obbard et al. 2012; Nolte et al. 2013). SNP frequencies were called using PoPoolation, which accounts for low frequency variants and variation in coverage that may influence results from pooled samples (Kofler et al. 2011). As found for *D. melanogaster*, AMPs have a higher mean Tajima’s *D* than background genes (−1.034 vs. −1.463). Linear models, as described above and in the Materials and Methods, revealed elevated Tajima’s *D* for AMPs in *D. mauritiana*, whereas Watterson’s *θ* and *π* were not significantly different when comparing AMPs and other genes in this species (fig. 1, table 1, and supplementary table S3, Supplementary Material online). Additionally, we again resampled the difference in these statistics between AMPs and neighboring control genes. We found AMPs have consistently higher values for *π*, Watterson’s *θ*, and Tajima’s *D* than their matched controls (fig. 1, table 2, and supplementary table S4, Supplementary Material online). For other immune genes, the differences from controls are primarily negative for *π*, Watterson’s *θ*, and Tajima’s *D*, suggesting directional selection may be acting on these genes in *D. mauritiana* (fig. 1 and supplementary tables S3 and S4, Supplementary Material online).

## Discussion

We find evidence consistent with balancing selection being an important evolutionary driver of AMP genes in *Drosophila*. This is most clearly observed in ancestral African populations (Zambia and Rwanda). There are several reasons why previous analyses may not have identified these selective forces acting on AMPs. First, signals of selection can be clouded by background selection. We found that the clearest signal for AMP balancing selection in the Rwandan population after using Comeron’s (2014) method to account for background selection. Second, previous studies have tended to group immune genes as a single entity when scanning genomes for footprints of selection. Strong directional selection acting on some receptor and signaling immune genes may swamp a subtler signal of balancing selection acting on AMPs. Third, this effect may be exacerbated by the fact that effector genes tend to be smaller (Lemaitre and Hoffmann 2007) than receptor and signaling genes, providing fewer sites and therefore less power to detect any population genetic signature. Relatedly, single gene analyses might lack the power to show such signatures. Fourth, patterns of nucleotide polymorphism are strongly influenced by population demographic history. Our AMP-control approach should account for the confounding influences of local mutation and recombination rate variation, gene size, and demography (Garrigan and Hedrick 2003).

The differences between derived (DGRP and France) and ancestral populations (Rwanda and Zambia) were striking. As populations establish in new habitats, they will encounter different pathogen pressures and prevailing environmental conditions. This could dramatically alter which alleles are selectively advantageous. First, bottlenecks may lead to the loss of one or more of the balanced alleles. Furthermore, loss of disadvantageous alleles (e.g., alleles resistant to pathogens not present in the new habitat) likely occurs more rapidly than establishment of new, beneficial polymorphisms (e.g., resistance alleles for newly encountered pathogens). This may explain why we find the strongest evidence for balancing selection on AMPs in ancestral African populations that have been coevolving with their pathogens, under semipredictable conditions, for long time-periods.

An alternate explanation for these differences could be that the quality of genomic data in the derived populations is lower than that for the ancestral populations (supplementary table S1, Supplementary Material online). We therefore limited our analysis to a subset of 114 DGRP lines with the highest quality data (DGPR-HQ, being those lines with fewest Ns and highest coverage, see Materials and Methods). Though we did still did not find strong evidence for balancing selection acting on AMPs in this population (DGRP-HQ in supplementary table S2, Supplementary Material online), both measures of diversity (pairwise diversity and Watterson’s theta) further increased in AMPs (but not other immune genes) when compared with the background, supporting an increase in diversity in AMPs compared with other genes. This also serves as a cautionary tale, as including lower quality genomes adds noise (increased standard deviation in resampling, supplementary table S2, Supplementary Material online) that could potentially mask population genetic signals.

It is tempting to look to newly developed methods for detecting balancing selection (e.g. DeGiorgio et al. 2014; Siewert and Voight 2017), but these statistics were developed for detecting the molecular footprints of selection in human populations. Assumptions about the genomic signatures of a balanced polymorphism that work well in humans are not applicable to *Drosophila*, because the window of linked polymorphism likely to show these signatures is tiny. To state this numerically, DeGiorgio et al. (2014), based on Gao et al. (2015), suggest a window size of 1/*ρ* (where *ρ* is the population-scaled recombination rate or 4*N*_e_*r*) for observing the signature of a linked balanced polymorphism. For humans, *ρ* is about 0.001 so the window size is about 1,000 bp (DeGiorgio et al. 2014). Estimates of *ρ* in *D. melanogaster* are highest in the DGRP population and range from 9.6 to 14.8 per kb for the different chromosomes (Chan et al. 2012). These values correspond to windows of 100 bp or less in *D. melanogaster*. Given estimates of nucleotide diversity between 0.001 and 0.01 (Lack et al. 2016), we expect less than one segregating site per window, rendering these tests unusable in this species. Recombination is even higher in *D. mauritiana* (True et al. 1996), and the use of pooled sequencing (Pool-seq) data for this species would further complicate the interpretation of newer selection statistics, due to the fact that low frequency alleles are dropped in Pool-seq data.

We find that, at least in ancestral populations, AMPs tend to evolve in a manner consistent with balancing selection, showing increased diversity but no increase in divergence (Unckless and Lazzaro 2016) relative to other genes. This contrasts with other immune genes which show no such pattern. Why are AMPs different than other immune genes? One characteristic of AMPs is that they interact directly with microbes (Bulet and Stocklin 2005), and, in some cases, AMP sequence is directly linked to the efficacy of bacterial membrane interactions (Schmitt et al. 2016; Franzoi et al. 2017). If particular AMP alleles encode for peptides that are more effective at fighting infection by particular microbes, a fluctuating suite of pathogens in the environment over time or space could lead to balanced polymorphisms. This “specificity hypothesis” suggests that allele frequencies in AMPs should vary spatially or temporally. There is some evidence for both seasonal (Behrman et al. 2018) and spatial (Early et al. 2017) variation in selection pressure on AMPs. However, evidence for AMP specificity against particular pathogens, especially different naturally occurring alleles of the same AMP, is currently rare (but see e.g., Tennessen et al. 2009; Hellgren et al. 2010; Park et al. 2014; Unckless et al. 2016). Additionally, the patterns of divergence and polymorphism expected after periods of fluctuating selection are not clear-cut and may complicate the detection of balancing selection (see, e.g., Huerta-Sanchez et al. 2008; Miura et al. 2013; Gossmann et al. 2014).

Alternatively, AMP variation might be maintained because AMP alleles that are more effective against pathogens also tend to carry a higher autoimmune cost. This “autoimmune hypothesis” states that more effective AMP alleles should be common during pathogen epidemics, but decrease in frequency when pathogens are rare. These patterns might also vary spatially and temporally, making the interpretation of these context-dependent patterns more difficult. There is evidence that overexpression of AMPs can have deleterious fitness consequences (Gilliet and Lande 2008; Benachour et al. 2009; Maneerat et al. 2016). However, it seems that if autoimmune costs were important in maintaining variation, we would also see signatures of balancing selection in the IMD and toll pathway signaling genes that control expression of AMPs. Most work suggests that these genes are evolving under directional selection, consistent with an arms race model (Obbard et al. 2006, 2009; Sackton et al. 2007). Distinguishing between these two hypotheses for the adaptive maintenance of AMP genetic variation will take careful functional analysis.

## Materials and Methods

### Polymorphism in Four Populations of *D. melanogaster*

We downloaded chromosome sequences for the Zambia (ZI, *n *=* *197), Rwanda (RG, *n *=* *27), *Drosophila* Genetic Reference Panel (DGRP, *n *=* *205), and France (FR, *n *=* *96) populations, available as part of the *Drosophila* Genome Nexus from http://www.johnpool.net/genomes.html; last accessed September 10, 2019. (Mackay et al. 2012; Pool et al. 2012). These data were collected as described elsewhere (Pool et al. 2012). Briefly, short read Illumina data were generated for each individual and mapped to the *D.melanogaster* reference genome version 5.22 using BWA and Stampy. Following mapping and alignment, GATK indel realigner was used to refine short indel alignments and SNPs were called for each individual. A custom reference genome was then generated for each individual based on all SNPs called and the process was repeated. Following a second round of mapping and indel realignment, SNPs called across both rounds were then inserted to create a second, final custom genome for each individual (Pool et al. 2012).

We converted these sequences into FASTA files, per chromosome, for each population. The RG and ZI populations are much higher quality data, the average per base coverage of the raw FASTQ data used to generate the FASTA files is much higher, and the number of ambiguous bases is much lower than the DGRP and FR populations (supplementary table S1, Supplementary Material online). Additionally, we created a subset of 114 DGRP lines (hereafter DGRP-HQ) with high coverage to calculate statistics in DGRP using only high-quality data (at least 25-fold coverage, and at least 75% of sites called in 1,000-bp windows across the genome).

Using annotation 5.57 of the *D. melanogaster* genome, we extracted FASTA alignments for each gene and extracted silent (four-fold degenerate) sites using a custom Biopython script (personal comm. Yasir Ahmed, May 2018). We then used a custom Python script with the package DendroPy, to find *π*, Watterson’s *θ*, Tajima’s *D*, and the number of segregating sites for the subset of silent sites per gene (Sukumaran and Holder 2010; Ferretti et al. 2012). We categorized genes involved in immune defense (hereafter immune genes) using the designations given at https://www.epfl.ch/labs/lemaitrelab/lemaitre-lab/resources/list-of-drosophila-genes-potentially-involved-in-the-immune-response/; last accessed September 10, 2019. (Lemaitre and Hoffmann 2007). We removed nonautosomal genes from all downstream analyses, because the X chromosome does not harbor any AMPs, and has a different effective population size than autosomes.

We employed a linear model in the form of *Y* = *chromosome + gene length + chromosome/region + chromosome/region/AMP*, where *Y* refers to the value of a particular statistic for that gene, region refers either to the region of the genome in which the gene is found (200,000-bp width) or a window around the focal AMP, and AMP refers to whether or not each gene was an AMP. Slashes such as in *chromosome/region* refer to *region* nested in *chromosome*. For the analysis of AMP silent polymorphism while accounting for background selection, we employed a linear model in the form of *Y* = *M1 *+* region/AMP* (Comeron 2014). All linear models included only genes on chromosomes 2L, 2R, and 3R, as these are the only chromosomes harboring AMPs in *D. melanogaster* and *D. mauritiana*. ANOVA results were analyzed using the *car* package (Fox and Weisberg 2011) in R v 3.5.1 (R Core Team 2017). The results of these models are summarized in supplementary table S3, Supplementary Material online.

To control for missing data, we removed all sequences containing over 25% ambiguous bases and recalculated each statistic using DendroPy v 4.4.0 in Python v 2.7.0, accounting for missing data in our calculation (Sukumaran and Holder 2010; Ferretti et al. 2012). We again fitted a linear model to this data, for each AMP and those control genes within 10,000, 50,000, and 100,000 bp of that AMP.

For each population, we then resampled to find the average difference in scores between AMP/immune genes and control genes. Genes were assigned as AMPs or immune genes based on data from Bruno Lemaitre. For each gene in these categories (AMP or immune), we randomly sampled a control gene in a 200,000-bp window centered on the AMP (100,000-bp upstream or downstream), that was no more than ten times larger than the focal gene (defined as coding sequence length measured in base pairs), and not another gene in the given category. We then found the average difference ($\overset{-}{\Delta }$) in each measure for the focal gene (AMP or immune) group and the control group such that

$$\;\overset{-}{\Delta }\;=\;\frac{1}{n}\;\sum_{i=1}^{n}(X\;\mathrm{AMP}/{\mathrm{Immune}}_{i}-X_{{\mathrm{Control}}_{i}}),$$

where *X* AMP/Immune represents the chosen AMP/immune gene, $X_{\mathrm{Control}}$ represents the randomly sampled control gene, and *n* accounts for the number of genes in the group. We then resampled 10,000 times to obtain an empirical distribution of the differences. Each AMP or immune gene was associated with between 8 and 47 control genes. With 10,000 resamplings, we expect only one particular combination of control genes to be chosen twice. We tested a range of window sizes (50,000–150,000 bp) and found qualitatively similar results (data not shown). To account for the fact that many AMPs cluster in the genome which might lead to pseudoreplication, we additionally repeated our analysis with a subsampled data set. To this end, we choose a subset of ten AMPs that were at least 5,000 bp from any other included AMPs and repeated the AMP-control analysis described above, with the specific subset of ten AMPs changing randomly per resampling.

We employed this method to control for genome-wide variation in recombination rates, mutation rates. Resampling 10,000 times allows for a robust empirical distribution that does not rely on the particular control genes chosen per iteration. We therefore present the distribution of differences as violin plots with the proportion of resamplings that do not overlap zero, analogous to a bootstrap value.

### Polymorphism in a Population of *D. mauritiana*

We downloaded the reference genome, annotation, and mapped BAM file of a population of *D. mauritiana* sequenced as a pool (Pool-seq) from http://www.popoolation.at/mauritiana_genome; last accessed September 10, 2019. (Nolte et al. 2013), and used PoPoolation to calculate *π*, Watterson’s *θ*, and Tajima’s *D* for each gene in this population. We then resampled to find the average difference in scores between AMPs/immune and a control set of genes, as described above.

### Data Availability

All data used in this study are publicly available and freely accessible. The *D. melanogaster* sequence data were obtained from John Pool’s *Drosophila* Genome Nexus (http://www.johnpool.net/genomes.html; last accessed September 10, 2019) in FASTA format. All *D. mauritiana* data (BAM file, reference genome and gene annotation) were downloaded from http://www.popoolation.at/mauritiana_genome; last accessed September 10, 2019.

## Supplementary Material

Supplementary data are available at *Genome Biology and Evolution* online.

## Supplementary Material

evz191_Supplementary_Data
