# Supplementary material for: Balancing Selection Drives the Maintenance of Genetic Variation in Drosophila Antimicrobial Peptides
Source: Genome Biol Evol. 2019 Sep 4;11(9):2691–701. doi: 10.1093/gbe/evz191 (PMC6764478; doi:10.1093/gbe/evz191)
Supplement: evz191_Supplementary_Data [file evz191_supplementary_data.zip › Supplementary Information Legends.docx]

**Supplementary Information**

**Supplementary Table S1** – **Quality of sequencing data used in this study.** Summary statistics for each *D. melanogaster* dataset, including the average base coverage for each population and the average number of ambiguous bases per 1000 bases in the FASTA files used. Data taken from <http://www.johnpool.net/genomes.html>.

**Supplementary Table S2** **- Summary statistics for each AMP, immune and control gene in five *D. melanogaster* datasets using only silent (four-fold degenerate) sites.** Also included are the FlyBase (<https://flybase.org/>) transcript identifiers for each gene; and, for AMP and immune genes, the gene length and number of matched control genes. The immune gene category excludes AMPs.

**Supplementary Table S3** **- Linear models for various population genetic statistics among five *Drosophila* populations suggests AMPs are elevated, consistent with balancing selection, in several instances.** Each value is the *F*-statistic and *P*-value from an ANOVA. *P*-values less than 0.05 are in bold. AMP refers to AMP nested in region nested in chromosome, and region refers to region nested in chromosome.

**Supplementary Table S4** - **Summary of resampling results for each AMP and its matched control genes using exclusively silent (four-fold degenerate) sites.** These statistics include the percentage of greater than 0, mean and standard deviation for each resampling set. Six *Drosophila* datasets from two species were analysed.

**Supplementary Figure S1** - **Tajima's D for each *D. melanogaster* population, for each AMP, compared to Tajima’s D for all control genes matched to that AMP.** Each grey dot is the selection of control genes within 100000 bp of the AMP, of a similar length. AMPs are shown in red, the median and interquartile range of the control genes are shown as a black dot and bar per AMP. Tajima’s D was calculated on silent (four-fold degenerate) sites only. DGRP = *Drosophila* Genetics Reference Panel from North Carolina, USA; FR = France; RG = Rwanda; ZI = Zambia.

**Supplementary Figure S2** – **Subsampling using only unlinked AMPs also suggests AMPs are subject to balancing selection in several cases.** Summary of resampling results (AMP - control) for AMPs for π (i.e. nucleotide diversity), Watterson's θ and Tajima's D, using randomly selected subsets of 10 non-linked AMPs. All three statistics (Tajima's D, π and θ_W_) were calculated on silent (four-fold degenerate) sites only. DGRP = *Drosophila* Genetics Reference Panel from North Carolina, USA; FR = France; RG = Rwanda; ZI = Zambia.
